# Structure and Wound-Healing Activity of a Branched Levan-Type Fructan from *Cyathula officinalis* Roots

**DOI:** 10.3390/molecules31111981

**Published:** 2026-06-05

**Authors:** Yujie Qiu, Chengcheng Cai, Lijuan Wu, Xinyi Zhao, Tianle Liu, Qingmiao Li, Sizhe Shui, Rui Li, Mengliang Tian, Hai Lan

**Affiliations:** 1 Key Laboratory of Biology and Genetic Improvement of Maize in Southwest Region, Maize Research Institute, Sichuan Agricultural University, Chengdu 611130, China; 2 College of Agronomy, Sichuan Agricultural University, Chengdu 611130, China; 3 Sichuan Key Laboratory of Traditional Chinese Medicine Quality Research and Innovation, Sichuan Academy of Chinese Medicine Sciences, Chengdu 610041, China; 4 Natural Medicine Research Center, College of Veterinary Medicine, Sichuan Agricultural University, Chengdu 611130, China

**Keywords:** *Cyathula officinalis*, CoPS, levan-type fructan, methylation–GC–MS, 1D/2D NMR spectroscopy, cutaneous wound repair

## Abstract

*Cyathula officinalis* Kuan, a medicinal plant used in traditional medicine, remains underexplored as a source of structurally defined wound-repair polysaccharides. In this study, a water-soluble polysaccharide fraction, CoPS, was isolated from *C. officinalis* roots and structurally characterized using methylation analysis and 1D/2D NMR spectroscopy. Purified CoPS had a total carbohydrate content of 94.8%, a weight-average molecular weight (*M*_w_) of 7.491 kDa, and a narrow dispersity (*M*_w_/*M*_n_ = 1.04). Monosaccharide composition analysis showed that CoPS was mainly composed of fructose and glucose at a molar ratio of 95.60:4.40. Structural analyses identified CoPS as a branched levan-type fructan with a β-(2→6)-linked fructofuranosyl backbone and β-(2→1)-linked branching motifs. CoPS was incorporated into a Carbomer/alginate topical formulation, termed CoPS-CPG, and evaluated in vitro and in vivo. CoPS-CPG showed good cytocompatibility and promoted HaCaT keratinocyte migration, reducing the residual scratch area to 48.10% at 12 h compared with 70.13% in the control group and 65.18% in the vehicle (Blank-CPG) group. In a murine full-thickness excisional wound model, CoPS-CPG reduced the residual wound area to 8.70 ± 1.20% on day 14, compared with 24.83 ± 1.51% in the control group and 14.20 ± 0.72% in the vehicle group. Histological and immunological analyses further indicated improved tissue reconstruction, a reduced inflammatory burden, enhanced CD206-associated macrophage signals, increased CD31-associated vascular structures, improved α-SMA-associated perivascular coverage, and lower late-stage HIF-1α expression. These findings identify CoPS as a structurally defined plant-derived levan-type fructan that supports cutaneous wound repair.

## 1. Introduction

Cutaneous wound healing is a highly coordinated process involving hemostasis, inflammation, proliferation, and remodeling [1,2]. Recent mechanistic studies further emphasize that effective skin repair requires the coordinated regulation of cell migration, proliferation, extracellular matrix deposition and remodeling, inflammation, and angiogenesis [2]. Under physiological conditions, repair is usually efficient; however, it is frequently impaired by diabetes, ischemia, infection, or excessive oxidative stress, resulting in chronic non-healing wounds that impose substantial clinical and socioeconomic burdens [3]. At the microenvironmental level, impaired repair is commonly associated with persistent inflammation and insufficient vascular reconstruction, which together limit oxygen and nutrient delivery and perpetuate tissue dysfunction [4,5]. In this context, hypoxia-responsive signaling provides an important interface between oxygen sensing and angiogenic remodeling by linking local oxygen tension to pro-angiogenic cues that support granulation tissue formation and regeneration [6,7,8]. Recent advances in wound dressings, including multifunctional hydrogel systems with antibiofilm and antioxidative properties, further highlight the need to regulate infection, oxidative stress, and the repair microenvironment simultaneously in difficult-to-heal wounds [9]. Accordingly, topical biomaterials and plant-derived biomolecules that coordinate inflammation resolution, epithelial repair, and vascular remodeling have attracted increasing interest for cutaneous repair.

Among plant-derived natural products, polysaccharides are particularly attractive because of their biocompatibility, biodegradability, and diverse bioactivities [10,11]. Natural products have been widely investigated for wound-healing applications, and many of their effects have been associated with antioxidant, anti-inflammatory, antimicrobial, and cell-migration-promoting activities [12]. Importantly, the biological properties of polysaccharides are strongly influenced by fine structural features, including monosaccharide composition, glycosidic linkages, molecular weight, and branching patterns. Detailed structural elucidation is therefore essential for understanding their biological functions and for establishing meaningful structure–activity relationships [13]. Structural characterization is particularly important when linking plant-derived polysaccharides to biologically meaningful wound-repair activity.

Fructans are polysaccharides composed predominantly of fructofuranosyl residues and represent a promising but comparatively underexplored group of plant-derived bioactive carbohydrates. According to their linkage patterns, fructans are mainly classified as inulin-type structures containing β-(2→1) linkages or levan-type structures containing β-(2→6) linkages [14,15]. Compared with the more extensively studied inulins, levan-type fructans, especially those with defined branching features, may have distinct physicochemical characteristics and immunomodulatory potential [16,17]. Nevertheless, structurally characterized plant-derived levan-type fructans remain relatively rare, and their therapeutic relevance in cutaneous wound repair is still insufficiently understood.

In the present study, we investigated whether a structurally resolved fructan fraction from *Cyathula officinalis* Kuan could support cutaneous repair after topical administration. We isolated a homogeneous water-soluble polysaccharide fraction (CoPS) from *C. officinalis* roots, elucidated its structure by methylation analysis and 1D/2D NMR spectroscopy, incorporated it into a Carbomer/alginate topical formulation, and evaluated its biological effects using cell-based assays and a murine excisional wound model. Our data identify CoPS as a branched levan-type fructan and associate topical CoPS-CPG treatment with accelerated macroscopic wound-area reduction, improved histological repair, reduced inflammatory burden, and vascular remodeling during healing.

## 2. Results

### 2.1. Physicochemical Properties and Preliminary Structural Characterization of CoPS

The crude polysaccharide yield was 34.2% on a dry-weight basis. Further purification afforded a purified fraction, termed CoPS, with a final yield of 1.8% relative to the dry weight of the starting material, equivalent to 5.3% recovery from the crude polysaccharide. Compositional analysis showed that CoPS was a neutral polysaccharide with a total carbohydrate content of 94.8%; proteins and phenolic compounds were not detected.

**Figure 1:**
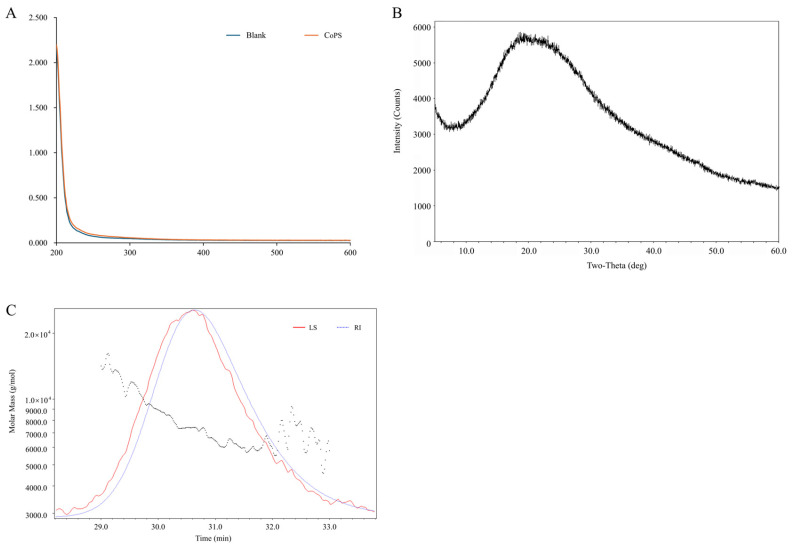
Physicochemical characterization of CoPS from *C. officinalis* roots. (**A**) UV-vis spectrum of CoPS. (**B**) X-ray diffraction pattern of CoPS. (**C**) SEC–MALLS–RI chromatogram and molar-mass distribution of CoPS.

We first characterized the basic physicochemical properties and preliminary structural features of CoPS. The UV-vis spectrum showed no distinct absorption peaks at 260 or 280 nm (Figure 1A). The A260 and A280 values were 0.081 and 0.068, respectively, with an A260/A280 ratio of 1.19. After blank subtraction, the net A260 and A280 values were only 0.018 and 0.015, respectively, indicating negligible nucleic acid/protein contamination in the purified CoPS fraction. Consistent with this purity profile, powder X-ray diffraction (XRD) analysis (Figure 1B) showed no sharp Bragg reflections; instead, the diffractogram displayed a broad amorphous halo with a maximum centered at approximately 20° (2θ). This pattern is characteristic of an amorphous non-crystalline solid state, which has been widely reported for fructans, including inulin- and levan-type materials, and related carbohydrate materials [18].

To further assess homogeneity and chain dimensions in solution, the purified polysaccharide was characterized by SEC–MALLS–RI (Figure 1C). CoPS exhibited *M*_n_ = 7.2 kDa, *M*_p_ = 7.401 kDa, *M*_w_ = 7.491 kDa, and *M*_z_ = 7.865 kDa, with a narrow dispersity (*M*_w_/*M*_n_) of 1.04, indicating a tight molecular-weight distribution and a highly uniform population. Notably, the refractive-index trace showed a single symmetric peak eluting at 29–33 min, and the reported recovery for this component accounted for essentially all collected mass, further supporting the presence of one dominant polysaccharide fraction rather than a mixture.

**Figure 2:**
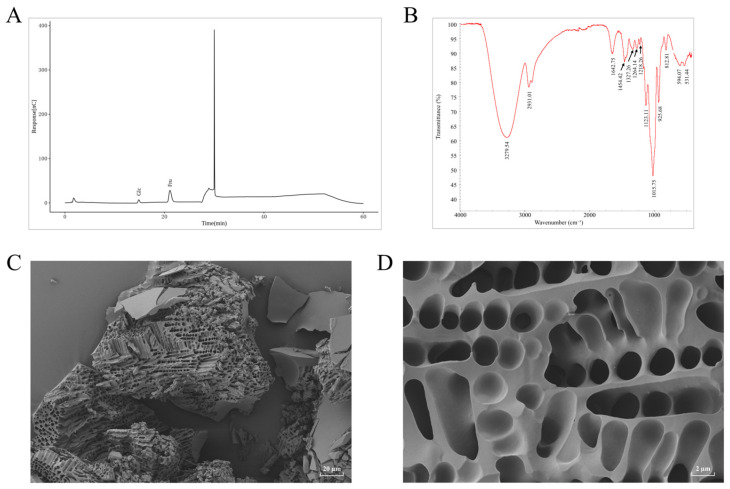
Structural and compositional analysis of CoPS from *C. officinalis* roots. (**A**) Monosaccharide composition determined by HPAEC–PAD. (**B**) FTIR spectrum of CoPS. (**C**,**D**) SEM images of CoPS showing porous microstructures at low and high magnifications. The images were acquired at 1000× (**C**) and 10,000× (**D**) magnification, with scale bars of 20 μm and 2 μm, respectively.

Monosaccharide composition analysis by HPAEC–PAD (Figure 2A) showed that the purified polysaccharide consisted predominantly of fructose (Fru, 95.60 mol%) with a minor proportion of glucose (Glc, 4.40 mol%). This Fru-dominant monosaccharide profile indicates that CoPS is a fructan, whereas the minor Glc fraction suggests that glucose may occur as a terminal residue associated with an initiating unit.

FTIR spectroscopy (Figure 2B) further corroborated the polysaccharide nature of CoPS and provided functional-group-level structural information. The broad band at 3279.54 cm^−1^ was attributed to O–H stretching vibrations from abundant hydroxyl groups and hydrogen bonding, while the band at 2931.01 cm^−1^ corresponded to C–H stretching of the carbohydrate framework. The absorption near 1642.75 cm^−1^ was consistent with O–H bending of bound/adsorbed water, a common feature of hydrophilic polysaccharides. In the fingerprint region, signals at 1327.26 and 1218.26 cm^−1^ were assigned to C–O–H bending and C–O–C stretching, whereas the strong bands at 1123.11 and 1015.75 cm^−1^ were assigned to C–O–C/C–O stretching vibrations of glycosidic linkages and furanosyl residues commonly observed in fructan-rich polysaccharides [19]. The bands around 925.68 and 812.81 cm^−1^ may be associated with ring vibrations and linkage-related modes, providing additional support for the fructan-rich nature of CoPS. Several weak bands in the lower wavenumber region (594.07, 531.44, and 424.99 cm^−1^) were also observed but were not considered diagnostic for carbohydrate backbone structure. SEM imaging (Figure 2C,D) revealed an uneven surface topography that was overall relatively smooth but notably porous, with abundant voids distributed across the surface. Such porous morphology may facilitate rehydration of the dried sample in aqueous media [20].

### 2.2. Glycosidic Linkage Analysis by Methylation–GC–MS

The glycosidic linkages of CoPS were characterized by methylation–GC–MS through identification of partially methylated alditol acetates (PMAAs). A representative GC–MS total ion chromatogram (TIC) of the PMAA derivatives is provided in Figure S1, with the major assigned peaks labeled according to the retention times listed in Table 1. Peak assignments were established based on relative retention behavior and EI–MS fragmentation patterns by comparison with published datasets and curated PMAA spectral resources [21,22]. Because fructose is a ketose, reduction of its carbonyl group during derivatization can yield two reduced isomers (glucitol- and mannitol-type), which may appear as paired GC peaks for the same fructose linkage class. Linkage-type distributions were therefore interpreted primarily using linkage subtotals. Because PMAA peak areas may be influenced by derivative-specific response factors and the ketose-derived paired-isomer formation of fructose, the methylation data were used primarily to infer linkage classes and relative linkage abundance rather than as an independent quantitative substitute for the HPAEC–PAD monosaccharide composition.

Based on normalized PMAA peak areas, Fru-derived PMAAs accounted for 89.53%, whereas Glc was detected exclusively as terminal glucopyranosyl residues (t-Glcp, 10.46%). Among the Fru-derived residues, 6-substituted fructofuranosyl residues (6-Fruf) formed the predominant linkage class (44.23%), suggesting that β-(2→6)-linked fructofuranosyl units constitute the major linkage type in CoPS and are consistent with a levan-type fructan backbone [23,24]. Terminal fructofuranosyl residues (t-Fruf) accounted for 25.99%, reflecting a relatively high abundance of chain ends. In addition, 1-substituted Fruf residues (1-Fruf, 7.67%) and 1,6-disubstituted fructofuranose residues (1,6-Fruf, 11.64%) were detected. These data are consistent with a β-(2→6)-dominated main chain containing branch points involving C-1 substitution and are compatible with the presence of β-(2→1)-linked side-chain motifs.

Taken together, the methylation–GC–MS results indicate that CoPS is a Fru-rich, branched fructan featuring a predominantly levan-type β-(2→6)-linked fructofuranosyl backbone, with branch points indicated by 1,6-disubstituted Fruf residues and Glc occurring as a terminal unit. Detailed structural features, including linkage configuration and residue assignments at the termini, are further supported by NMR analysis.

### 2.3. Detailed Structural Elucidation by 1D/2D NMR Spectroscopy

**Figure 3:**
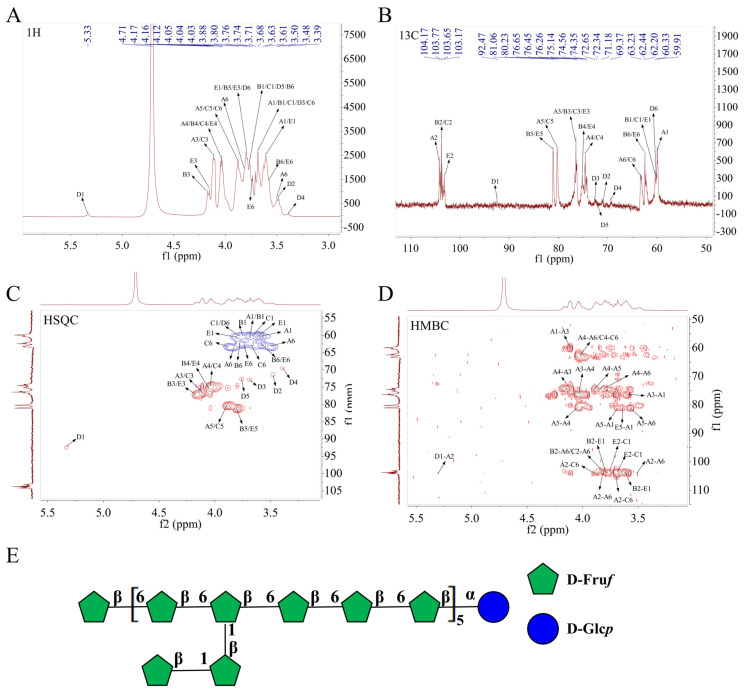
NMR-based structural elucidation of CoPS. (**A**) ^1^H NMR spectrum. (**B**) ^13^C NMR spectrum. (**C**) HSQC spectrum. (**D**) HMBC spectrum. (**E**) Proposed structural model of CoPS. The blue numbers in panels A and B indicate the chemical shift values (ppm) of the corresponding NMR signals.

To corroborate the linkage features inferred from methylation–GC–MS and resolve residue-level configurations, CoPS was further characterized by 1D (^1^H, ^13^C, and DEPT-135) and 2D (COSY, TOCSY, HSQC, and HMBC) NMR spectroscopy. In the ^1^H NMR spectrum, the anomeric region (δH 4.4–5.3 ppm) was essentially featureless, aside from the HOD signal at δH 4.71 ppm. This feature is characteristic of fructan-rich polysaccharides because the “anomeric” carbon of fructofuranosyl residues is C-2, which is quaternary and does not carry a directly attached proton. A weak resonance at δH 5.33 ppm was observed and assigned to an α-glucopyranosyl residue (residue D). The remaining proton signals were mainly distributed over δH 3.1–4.3 ppm with substantial overlap, necessitating 2D experiments for reliable assignment (Figure 3A).

In the ^13^C NMR spectrum, four prominent resonances in the fructan C-2 region (δC 103–104 ppm) were detected at δC 104.17, 103.65, 103.77, and 103.17 ppm and designated as residues A, B, C, and E, respectively (Figure 3B). Consistently, HSQC provided the corresponding ^1^H/^13^C one-bond correlations; an anomeric cross-peak assignable to residue D was observed at δH/δC 5.33/92.47 ppm (H-1/C-1), whereas, as expected, no direct HSQC correlations were observed for the quaternary C-2 signals of residues A, B, C, and E. DEPT-135 further revealed multiple methylene carbon signals in the range of δC 59.91–63.23 ppm, consistent with C-1/C-6 environments of fructofuranosyl residues and C-6 of glucopyranose.

Residue assignments were established by integrating HSQC data (Figure 3C), COSY and TOCSY spectra (Figures S2 and S3), diagnostic fructan chemical-shift patterns, and linkage information obtained from methylation analysis. Accordingly, residue A was assigned to →6)-β-D-Fruf-(2→, residue B to terminal β-D-Fruf-(2→, residue C to →1,6)-β-D-Fruf-(2→ (branching residue), residue E to →1)-β-D-Fruf-(2→, and residue D to α-D-Glcp-(1→. The complete ^1^H/^13^C chemical-shift assignments are summarized in Table 2. Inter-residue connectivities were subsequently verified by HMBC correlations. A series of long-range correlations between fructofuranosyl C-2 signals and acceptor H-6 protons were observed, including C-2(A)/H-6(A) at δC/δH 104.17/3.82 (3.50) ppm and C-2(A)/H-6(C) at 104.17/3.87 (3.68) ppm, as well as C-2(B)/H-6(A) at 103.65/3.82 ppm and C-2(C)/H-6(A) at 103.77/3.82 ppm. These C-2→H-6 correlations are diagnostic of β-(2→6) fructosyl linkages and therefore support a levan-type β-(2→6)-linked fructofuranosyl backbone. In addition, C-2(B)/H-1(E) at 103.65/3.81 (3.62) ppm and C-2(E)/H-1(C) at 103.17/3.77 (3.67) ppm provided direct evidence for C-2→H-1 connectivities, supporting the presence of β-(2→1) linkages associated with the →1)-substituted residue E and the →1,6)-disubstituted residue C. Finally, an HMBC cross-peak between H-1(D) and C-2(A) (δH/δC 5.33/104.17 ppm) established the glucosyl-to-fructosyl linkage at the terminal motif, consistent with an α-D-Glcp-(1→2)-β-D-Fruf initiating unit (Figure 3D).

Collectively, the NMR data define CoPS as a branched levan-type fructan. The main chain is dominated by β-(2→6)-linked fructofuranosyl residues (→6)-β-D-Fruf-(2→), with branching points represented by →1,6)-β-D-Fruf-(2→ residues. The side-chain motif involves β-(2→1)-linked fructofuranosyl units attached at O-1 of the branching residues, and a terminal α-D-Glcp residue is present in the end-group region. This NMR-defined architecture is fully consistent with the methylation–GC–MS linkage distribution, which showed predominant 6-substituted Fruf together with 1,6-disubstituted Fruf and terminal residues, jointly supporting a Fru-rich, β-(2→6)-dominated branched fructan structure for CoPS. A schematic representation of the inferred polysaccharide architecture is shown in Figure 3E.

### 2.4. Appearance, Rheological Behavior, and pH Stability of the Topical Formulation

**Figure 4:**
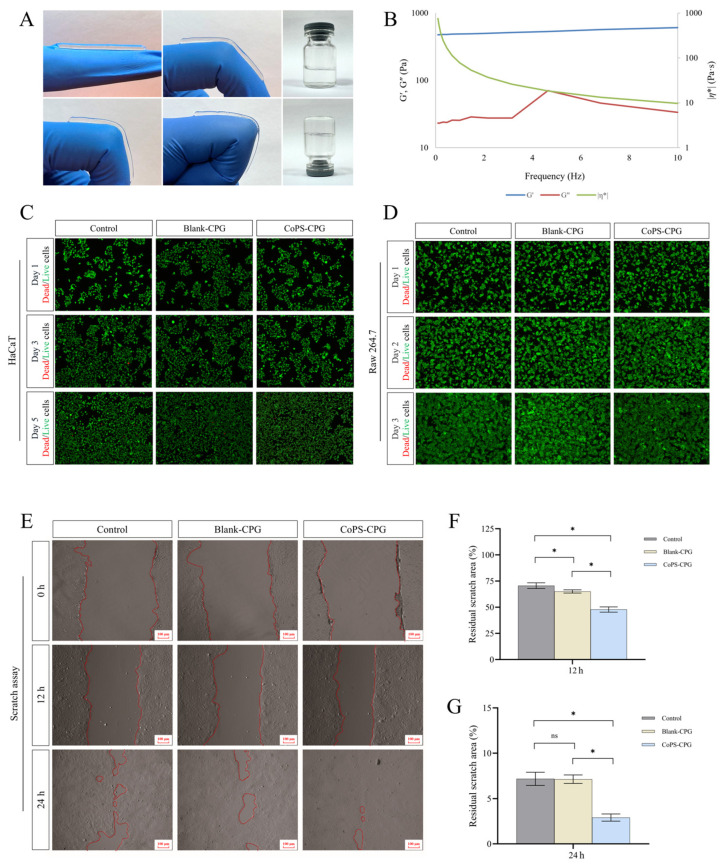
Preliminary formulation characteristics, cytocompatibility, and in vitro migration analysis of CoPS-CPG. (**A**) Representative photographs showing the appearance, spreadability, and semisolid character of CoPS-CPG. (**B**) Oscillatory rheological behavior of CoPS-CPG, showing storage modulus (G′), loss modulus (G″), and complex viscosity |η*| as a function of frequency. (**C**,**D**) Representative live/dead staining im-ages of HaCaT and RAW264.7 cells cultured with TCP, Blank-CPG, or CoPS-CPG. (**E**) Representative scratch images of HaCaT cells at 0, 12, and 24 h. Scale bar: 100 μm. (**F**,**G**) Quantification of the residual scratch area at 12 and 24 h.

As shown in Figure 4A, the CoPS-containing formulation exhibited a smooth and homogeneous appearance. It could be spread uniformly on the glove surface and remained in place under simple inversion conditions, indicating a semisolid consistency suitable for topical application. To further evaluate its basic formulation properties, oscillatory rheological analysis was performed over a frequency range of 0.1–10 Hz. As shown in Figure 4B, the storage modulus (G′) was consistently higher than the loss modulus (G″) throughout the tested frequency range, with G′ ranging from 475.88 to 606.49 Pa and G″ ranging from 23.08 to 70.68 Pa. The corresponding tan δ values remained below 1, indicating an elastic-dominant gel network. In addition, the complex viscosity |η*| decreased from 758.28 Pa·s at 0.1 Hz to 9.67 Pa·s at 10 Hz, suggesting frequency-dependent viscosity reduction under dynamic deformation. This behavior may facilitate spreading during application while supporting retention under low-deformation conditions.

Short-term pH and preliminary physical stability were further evaluated over 7 days (Table S1). The pH values of Blank-CPG and CoPS-CPG remained within a narrow range of 6.47–6.51 during the observation period, with only minor changes from day 0 to day 7. Both formulations maintained a homogeneous appearance and semisolid consistency, without visible phase separation, precipitation, discoloration, or obvious loss of consistency. These results indicate that CoPS incorporation did not adversely affect the short-term pH or physical stability of the Carbomer/alginate formulation. Together, the appearance, rheological, and stability data support the practical applicability of CoPS-CPG as a topical semisolid formulation.

### 2.5. Cytocompatibility of the Vehicle and CoPS-Containing Formulations in HaCaT and RAW264.7 Cells

Before subsequent biological evaluation, the cytocompatibility of Blank-CPG and CoPS-CPG was assessed in HaCaT keratinocytes and RAW264.7 macrophages. Live/dead staining showed that HaCaT cells cultured on formulation-coated surfaces exhibited predominantly calcein-AM-positive (green) signals, with only minimal PI-positive (red) cells, comparable to the tissue culture polystyrene (TCP) control (Figure 4C). Similar staining patterns were observed in RAW264.7 cells under the same conditions (Figure 4D). Quantitative analysis further showed that cell viability remained high across all groups, with no obvious reduction after exposure to either Blank-CPG or CoPS-CPG. In addition, both cell types maintained normal adherent morphology and relatively uniform distribution, indicating that neither the vehicle formulation nor CoPS incorporation caused apparent cytotoxicity under the present experimental conditions. These results support the cytocompatibility of the formulation system for subsequent in vitro and in vivo evaluation.

### 2.6. CoPS-CPG Promotes Keratinocyte Migration In Vitro

Keratinocyte migration is a critical step in re-epithelialization and is widely used as an in vitro indicator of the pro-healing potential of biomaterials [25]. We therefore evaluated whether CoPS-CPG could enhance keratinocyte migration using a scratch assay in HaCaT monolayers under serum-reduced conditions to limit proliferation-driven closure (Figure 4E). At 12 h, the residual scratch area decreased to 48.10% in the CoPS-CPG group, compared with 70.13% in the control group and 65.18% in the Blank-CPG group. By 24 h, only a small residual scratch area remained in the CoPS-CPG group, whereas larger open areas were still observed in both control groups (Figure 4F,G). These findings indicate that CoPS-CPG promoted HaCaT cell migration in vitro.

### 2.7. Topical CoPS-CPG Was Associated with Reduced Residual Wound Area and Improved Histological Repair In Vivo

**Figure 5:**
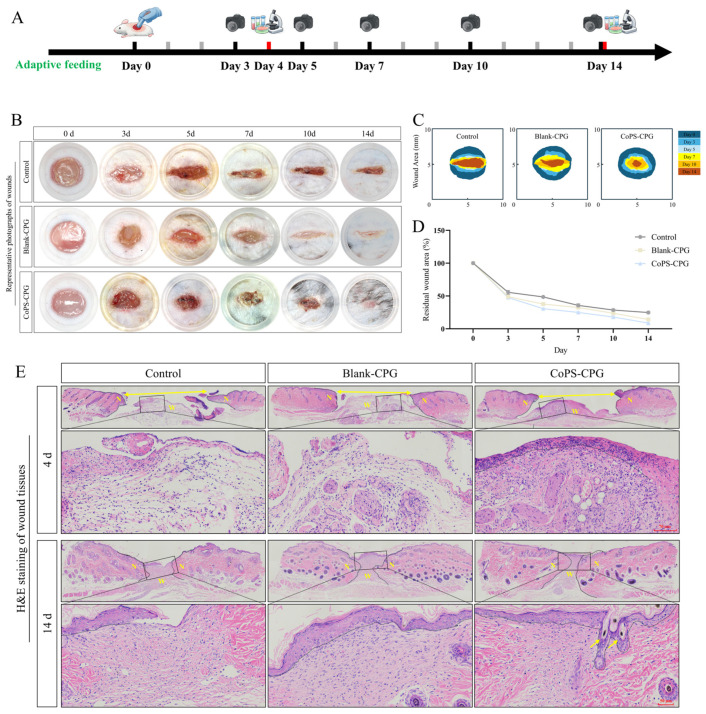
Effect of topical CoPS-CPG on residual wound-area reduction and histological repair in a murine full-thickness excisional wound model. (**A**) Experimental design. In panel A, adaptive feeding indicates the acclimatization period before Day 0; camera icons indicate wound imaging, tube/microscope icons indicate sample collection and subsequent analysis, and red ticks indicate tissue-collection time points. (**B**) Representative wound photographs obtained on days 0, 3, 5, 7, 10, and 14. (**C**) Time-course of normalized wound area. (**D**) Quantification of residual wound area over time, expressed as a percentage of the initial wound area on day 0. Data are presented as mean ± SD, *n* = 6 per group, analyzed by two-way repeated-measures ANOVA followed by Tukey’s post hoc test. (**E**) Representative H&E images of wound tissues collected on days 4 and 14. Scale bars: 70 μm for day 4 images and 50 μm for day 14 images (4× objective magnification; digitally cropped/enlarged views were generated from the same original images). In the image, N and W indicate normal tissue and the wound area, respectively; yellow double-headed arrows indicate wound width, and yellow arrows indicate regenerated skin appendages.

To evaluate the effect of topical treatment on cutaneous wound repair, we monitored macroscopic wound-area changes over time in a murine full-thickness excisional wound model. The experimental workflow is shown in Figure 5A. Representative macroscopic images were recorded on days 0, 3, 5, 7, 10, and 14 (Figure 5B). All groups exhibited comparable initial wound areas on day 0, indicating good baseline consistency. As healing progressed, the residual wound area gradually decreased in all groups, although the rate of reduction differed among treatments (Figure 5C,D). The control group showed a slower reduction in residual wound area, with a visible residual open wound/eschar area still present on day 14. The Blank-CPG group showed a smaller residual wound area than the control group during the later stage of healing. In comparison, the CoPS-CPG group showed a more rapid decrease in residual wound area and a smaller eschar area during the early-to-mid stage (days 3–7). By day 10, residual wound-area reduction was more pronounced in the CoPS-CPG group, and by day 14, the treated wounds showed only minimal residual open areas. Apparent hair regrowth was also observed in the regenerated area of some CoPS-CPG-treated wounds on day 14.

Because the present murine excisional wounds were not splinted and may heal through a combination of contraction, epithelial coverage, granulation tissue formation, and remodeling, macroscopic wound-area reduction alone cannot distinguish the relative contributions of these processes. Therefore, H&E staining was performed to further evaluate epithelial coverage and tissue reconstruction on days 4 and 14 (Figure 5E). On day 4, the control group showed an obvious central tissue defect, limited epithelial advancement from the wound margins, loose wound-bed organization, and substantial inflammatory cell infiltration. The Blank-CPG group displayed partial improvement in tissue filling and wound-bed organization. In contrast, the CoPS-CPG group showed more advanced epithelial extension toward the wound center, together with denser granulation-like tissue and relatively reduced inflammatory infiltration. On day 14, although control wounds were largely covered, tissue organization remained incomplete, and adnexal restoration was limited. The Blank-CPG group showed more complete epithelial coverage. Notably, the CoPS-CPG group exhibited a more continuous epidermal layer and tissue architecture more closely resembling normal skin. Follicular/adnexal-like structures containing hair-shaft-like keratinized cores were also observed in the regenerated dermis, suggesting improved histological repair quality in the CoPS-CPG-treated group. To further support this morphological observation, hair follicle-like/adnexal-like structures were semi-quantitatively counted in day-14 H&E-stained sections. The number of follicle/adnexal-like structures per field of view was higher in the CoPS-CPG group than in the control and Blank-CPG groups. Specifically, CoPS-CPG-treated wounds showed 2.80 ± 0.84 structures per field, compared with 0.40 ± 0.55 in the control group and 0.60 ± 0.55 in the Blank-CPG group (Table S2). These results provide additional morphological support for improved repair quality after CoPS-CPG treatment. Because the analysis was based on H&E morphology, these structures were interpreted as follicle/adnexal-like structures rather than definitive regenerated hair follicles.

### 2.8. CoPS-CPG Was Associated with Reduced Inflammatory Burden and Changes in Macrophage-Associated Signals

**Figure 6:**
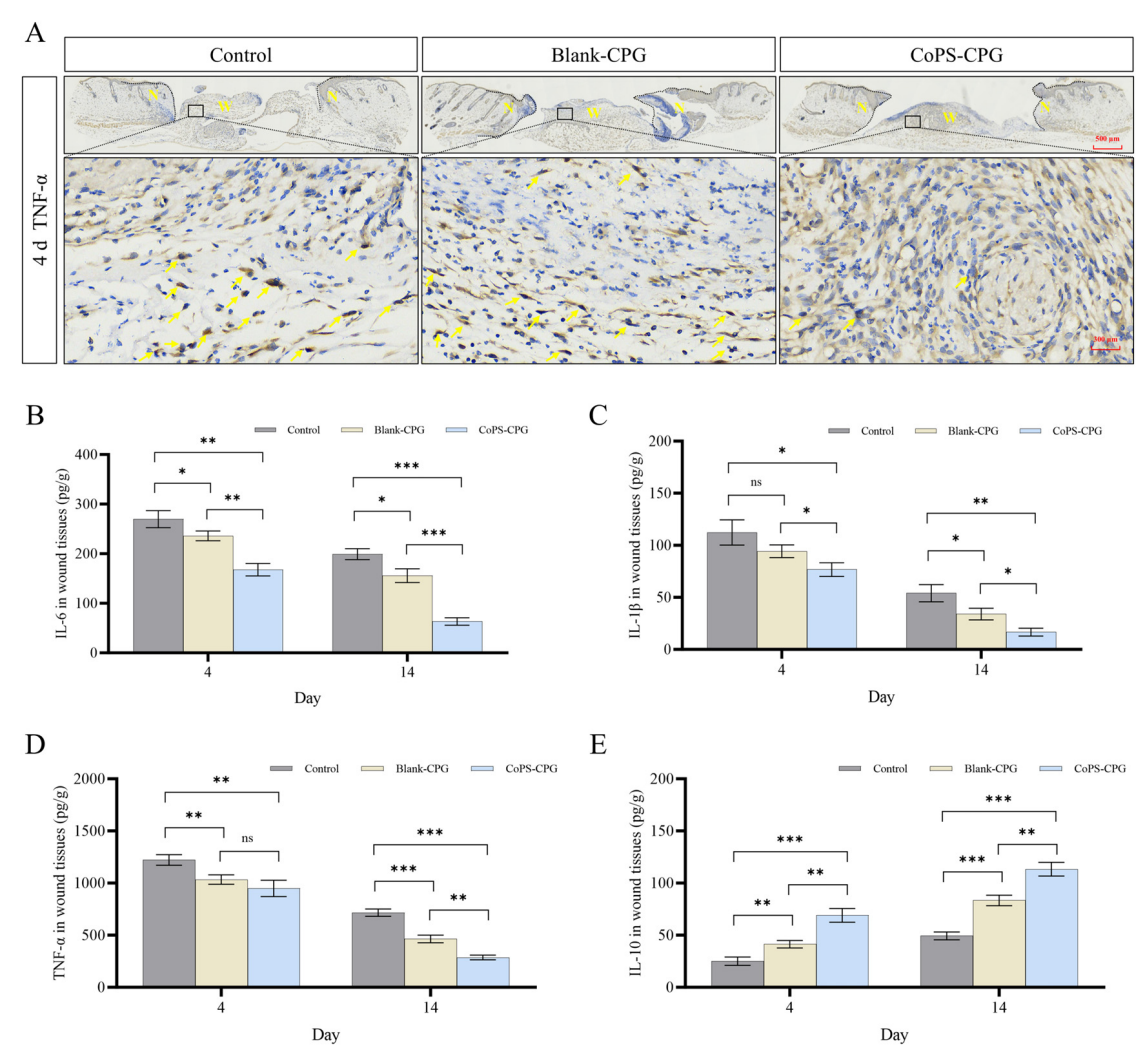
Inflammation-related markers in wounds treated with CoPS-CPG. (**A**) Representative immunohistochemistry images showing TNF-α expression in wound tissues on day 4. In the image, N and W indicate normal tissue and the wound area, respectively; yellow arrows indicate representative TNF-α-positive staining areas. Scale bars: 500 μm and 300 μm (10× objective magnification; digitally cropped/enlarged views were generated from the same original images). (**B**–**E**) Quantification of IL-6, IL-1β, TNF-α, and IL-10 levels in wound tissue homogenates on days 4 and 14.

Because excessive or prolonged inflammation is a major impediment to wound healing, we first evaluated the inflammatory status of wounds during the early stage of repair. TNF-α, a representative pro-inflammatory cytokine, was examined by immunohistochemistry on day 4 (Figure 6A). The control group showed strong positive staining in the wound bed and wound margins, consistent with an active local inflammatory response. In comparison, TNF-α positivity was reduced in the CoPS-CPG group, suggesting a lower local inflammatory burden.

To further assess inflammation-related changes at the tissue level, representative cytokines, including IL-6, IL-1β, TNF-α, and IL-10, were quantified in wound tissue homogenates on days 4 and 14 by ELISA (Figure 6B–E). Consistent with the TNF-α immunohistochemical findings, CoPS-CPG treatment was associated with lower levels of the pro-inflammatory cytokines IL-6, IL-1β, and TNF-α, together with increased IL-10 levels. On day 14, the overall inflammatory burden remained lower in the CoPS-CPG group. These findings were consistent with a more repair-associated cytokine profile during wound healing.

**Figure 7:**
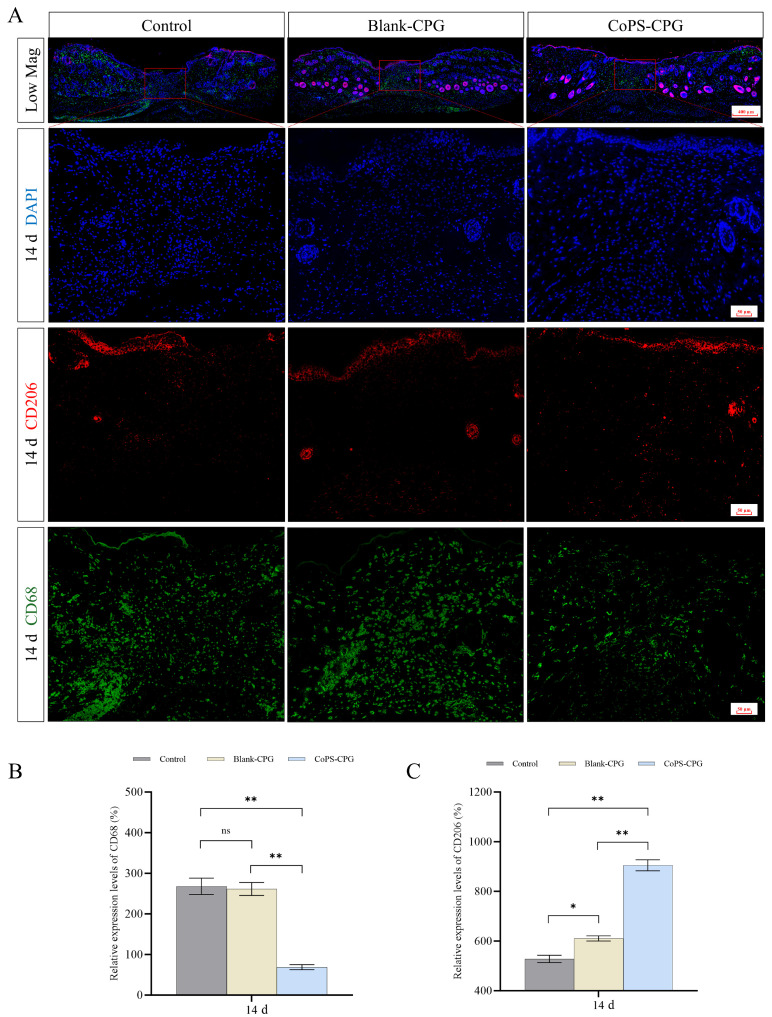
Macrophage phenotype and CD68/CD206 expression in wounds treated with CoPS-CPG. (**A**) Representative CD68/CD206 double immunofluorescence images on day 14. Scale bars: 400 μm and 50 μm (10× objective magnification; digitally cropped/enlarged views were generated from the same original images). (**B**,**C**) Quantification of CD68 and CD206 expression at the wound site.

Because macrophage-associated responses are closely related to inflammation resolution and tissue remodeling, CD68/CD206 double immunofluorescence staining was performed on day 14 to further characterize the immune microenvironment (Figure 7A). In the control group, numerous CD68^+^ cells were observed in the wound area, whereas the CD206 signal was relatively weak and showed limited overlap with CD68. In contrast, the CoPS-CPG group showed increased CD206-associated signal and greater CD68/CD206 co-localization, whereas the overall CD68 signal tended to decrease (Figure 7B,C). Together, these observations were consistent with reduced inflammatory cell burden and enrichment of CD206-associated macrophage features in the treated wounds. Notably, although CD206 is commonly used as a marker associated with M2-like or reparative macrophage programs, macrophage phenotypes are heterogeneous and cannot be conclusively defined by a single marker [26,27]. Therefore, the CD68/CD206 staining results should be interpreted as indicative evidence of a repair-associated macrophage shift rather than definitive proof of M1-to-M2 polarization. Overall, these findings suggest that CoPS-CPG treatment was associated with a more inflammation-resolved and repair-associated immune profile.

### 2.9. CoPS-CPG Was Associated with Enhanced Proliferation and Improved Vascular Organization During Healing

**Figure 8:**
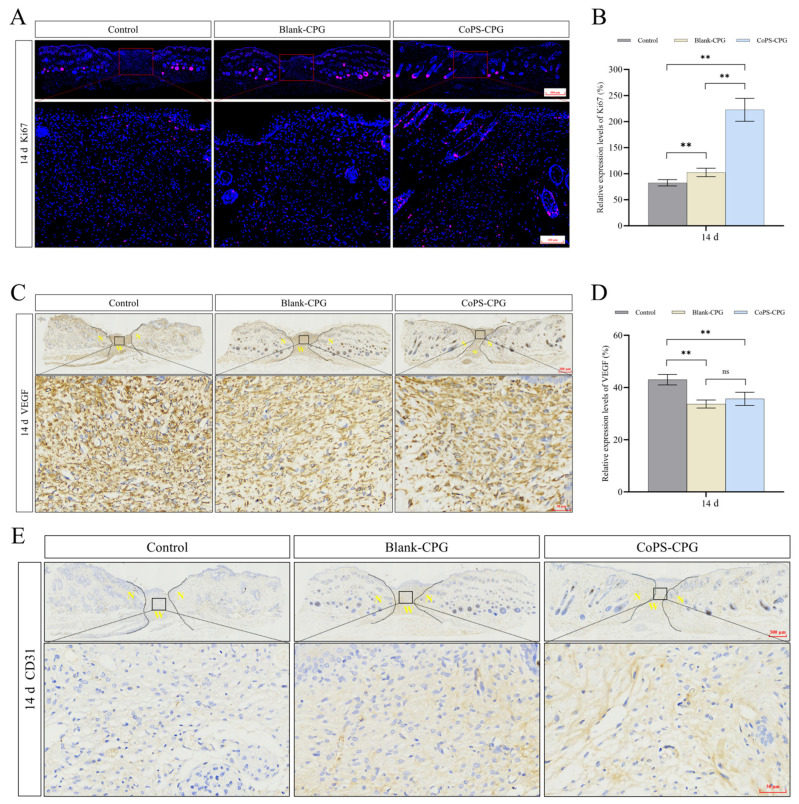
Proliferation and vascular organization-associated markers in CoPS-CPG-treated wounds. (**A**) Representative Ki67 immunofluorescence images showing proliferating cells in wound tissue on day 14. Scale bars: 500 μm and 100 μm (10× objective magnification; digitally cropped/enlarged views were generated from the same original images). (**B**) Quantification of Ki67-positive cells within the wound area. (**C**) Representative VEGF immunohistochemistry images showing VEGF expression in wound tissues on day 14. Scale bars: 300 μm and 30 μm (10× objective magnification; digitally cropped/enlarged views were generated from the same original images). (**D**) Quantification of VEGF-positive area in wound tissues on day 14. (**E**) Representative CD31 immunohistochemistry images indicating vascular structures in wound tissues on day 14. Scale bars: 300 μm and 30 μm (10× objective magnification; digitally cropped/enlarged views were generated from the same original images). Data are presented as mean ± SD, *n* = 6 per group, analyzed by one-way ANOVA followed by Tukey’s post hoc test.

Because active cell proliferation contributes to tissue filling and re-epithelialization during wound repair, proliferative activity within the wound bed was evaluated by Ki67 immunofluorescence staining on day 14 (Figure 8A,B). In the control group, only a limited number of Ki67-positive cells were observed at the wound margin and within the granulation tissue, mainly in the basal layer of the epidermis. In contrast, the CoPS-CPG group showed a stronger Ki67-positive signal, with a greater number of positive cells and a broader distribution at the re-epithelialization front and within the newly formed tissue area. This finding was consistent with the more continuous epidermal layer and improved histological architecture observed in the H&E sections, suggesting sustained proliferative activity in the treated wounds at this stage.

Because VEGF is a key pro-angiogenic and hypoxia-responsive factor during wound repair, VEGF expression was examined in wound tissues on day 14. Quantitative analysis of VEGF immunohistochemistry showed that the VEGF-positive area was highest in the control group and significantly lower in both the Blank-CPG and CoPS-CPG groups, whereas no significant difference was detected between the Blank-CPG and CoPS-CPG groups (Figure 8C,D). Thus, VEGF expression on day 14 did not indicate sustained VEGF upregulation after CoPS-CPG treatment. Instead, considering the increased CD31-positive vascular structures (Figure 8E), enhanced α-SMA-associated perivascular coverage, and reduced HIF-1α signal observed in CoPS-CPG-treated wounds, the day-14 VEGF result may reflect a more advanced repair stage with reduced hypoxia-driven VEGF demand.

**Figure 9:**
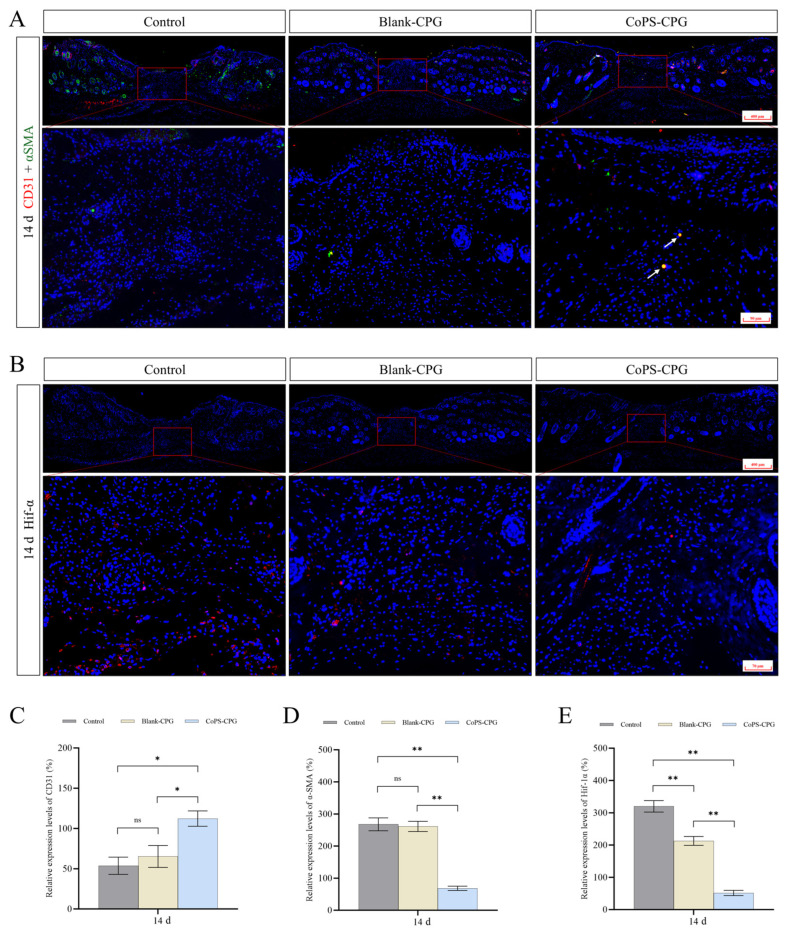
Perivascular coverage, vascular maturation-associated features, and hypoxia-related signals in CoPS-CPG-treated wounds. (**A**) Representative CD31/α-SMA double immunofluorescence images showing vascular structures and perivascular coverage in wound tissues on day 14. White arrows indicate CD31/α-SMA colocalization. Scale bars: 400 μm and 90 μm (10× objective magnification; digitally cropped/enlarged views were generated from the same original images). (**B**) Representative HIF-1α immunofluorescence images indicating hypoxia-related signals in wound tissue on day 14. Scale bars: 400 μm and 70 μm (10× objective magnification; digitally cropped/enlarged views were generated from the same original images). (**C**) Quantification of CD31 expression in wound tissues. (**D**) Quantification of α-SMA expression in wound tissues. (**E**) Quantification of HIF-1α expression in wound tissues. Data are presented as mean ± SD, *n* = 6 per group, analyzed by one-way ANOVA followed by Tukey’s post hoc test.

To further evaluate vessel-associated organization, CD31/α-SMA double immunofluorescence staining was performed on day 14 (Figure 9A). In the control group, CD31-positive vascular structures were relatively sparse and showed weak or discontinuous α-SMA coverage, with limited spatial overlap between the two signals. In contrast, the CoPS-CPG group exhibited a greater abundance of CD31-positive vascular structures together with a more continuous ring- or band-like α-SMA pattern closely apposed to the endothelial signal. Quantification of CD31 (Figure 9C) and α-SMA fluorescence (Figure 9D) was consistent with increased vascular structures and enhanced α-SMA-associated perivascular coverage in the treated group. Together with the closer spatial apposition of α-SMA signals to CD31-positive endothelial structures, these findings suggest a more stabilized and maturation-associated vascular phenotype in CoPS-CPG-treated wounds compared with the control group.

To assess hypoxia-related changes in the regenerated tissue, HIF-1α immunofluorescence staining was examined on day 14 (Figure 9B). HIF-1α signal remained strong in control wounds, whereas the CoPS-CPG group showed reduced HIF-1α intensity and distribution. Quantification of HIF-1α fluorescence (Figure 9E) was consistent with a lower hypoxia-related signal in the treated group at this stage of healing.

## 3. Discussion

Resolving CoPS as a low-molecular-weight branched levan provides a useful basis for discussing its potential biological activity, because it allows this polysaccharide to be considered as a chemically defined bioactive fructan rather than as a poorly characterized polysaccharide mixture [28]. The identification of a β-(2→6)-linked fructofuranosyl backbone with β-(2→1)-linked branches supports a structure–function perspective in which linkage pattern, branching features, and molecular size may influence biological responses. Previous studies on bioactive polysaccharides have shown that innate immune recognition is highly context dependent and that carbohydrate epitope characteristics and interfacial presentation can affect pattern-recognition-receptor-associated signaling [29,30]. In this regard, the defined fructan architecture of CoPS may be relevant to its observed wound-healing-related activity. In addition, its relatively low molecular weight and narrow dispersity may favor mobility and availability in hydrated topical formulations and wound exudates, thereby facilitating interactions with cells in the wound microenvironment [31,32,33]. Although these possibilities were not directly examined in the present study, the structural features of CoPS provide a plausible basis for its biological effects during cutaneous wound repair.

The biological findings further suggest that the CoPS-containing formulation may promote progression from the inflammatory phase toward tissue repair, at least in part through modulation of inflammation-associated and macrophage-related responses [34,35]. Excessive or prolonged TNF-α signaling is a characteristic feature of non-resolving inflammation and is often associated with impaired transition to the proliferative phase of wound healing. In this study, reduced TNF-α immunoreactivity in the CoPS-CPG group suggests that topical administration of the CoPS-containing formulation was associated with attenuation of local inflammatory burden, which may have contributed to a more permissive environment for granulation tissue formation and re-epithelialization. Macrophage responses during wound healing are temporally dynamic, with early inflammation-associated activities supporting debris clearance and later pro-resolution states contributing to extracellular matrix remodeling and tissue repair [5,36]. Consistent with this pattern, increased CD206/CD68 co-localization in regenerated tissue suggests enrichment of repair-associated macrophage features following CoPS-CPG treatment [37]. Although receptor engagement and downstream signaling pathways were not directly investigated, it remains biologically plausible that a structurally defined fructan may influence immune-related signaling. Taken together, the concurrent reduction in TNF-α signal and enrichment of CD206-associated macrophage features may help explain the improved tissue architecture observed at later stages in the CoPS-CPG-treated group.

The vascular effects observed in the CoPS-CPG-treated group appeared to involve increased endothelial-associated signals and enhanced perivascular coverage, which may reflect a more effectively resolved hypoxia–angiogenesis response during healing. Productive wound repair requires not only capillary sprouting but also maturation of newly formed microvessels into stable and functional vascular structures [38]. In the present study, VEGF expression was quantified on day 14, and the VEGF-positive area was not increased in the CoPS-CPG group. Instead, VEGF positivity was lower than that in the control group and showed no significant difference from the Blank-CPG group. Therefore, day-14 VEGF staining does not support sustained VEGF upregulation after CoPS-CPG treatment. However, increased CD31^+^ microvascular structures together with enhanced α-SMA-associated perivascular coverage around CD31^+^ vessels suggest that CoPS-CPG treatment was associated with improved vascular organization and a more maturation-associated vascular phenotype, rather than persistent VEGF elevation. On day 14, α-SMA signals showed ring- or band-like perivascular patterns closely apposed to CD31^+^ endothelial structures, consistent with improved vessel coverage and a more mature vascular organization compared with control wounds. Such structural organization is generally considered indicative of vascular stabilization and may contribute to better microcirculatory function during tissue remodeling [39,40]. Notably, the lower HIF-1α signal observed on day 14 in the CoPS-CPG-treated group should not necessarily be interpreted as reduced angiogenic repair; rather, it may be consistent with improved perfusion and oxygenation at a later stage of repair [41,42]. In wounds with delayed healing, persistent hypoxia is often accompanied by sustained HIF-1α elevation, reflecting an unresolved oxygen deficit. Therefore, the coexistence of increased CD31/α-SMA-associated vascular organization, reduced late-stage HIF-1α, and lower VEGF expression on day 14 may indicate a transition toward a more maturation-associated vascular organization with reduced late-stage hypoxia-related signaling with reduced hypoxia-driven VEGF demand. Nevertheless, because α-SMA can also be expressed by myofibroblasts in the wound bed and additional mural-cell markers or direct perfusion assays were not included, these findings should be interpreted as maturation-associated evidence rather than definitive proof of fully functional vascular maturation. Moreover, because VEGF was examined only on day 14, the present data cannot determine whether CoPS-CPG altered earlier VEGF dynamics during the proliferative phase of wound healing.

By integrating inflammation-associated improvement with vascular reconstruction, the CoPS-containing formulation may help establish conditions that are more favorable for tissue rebuilding during the later stages of repair. The increased Ki67 signal detected at the wound edge and within regenerated tissue suggests that proliferative activity was maintained in the treated group during a period when control wounds showed lower cycling activity, implying that CoPS-CPG treatment may support a more active reparative state [36,43]. This observation is consistent with the concept that keratinocyte and fibroblast proliferation is closely influenced by inflammatory tone and oxygen/nutrient supply [42]. In this context, attenuation of excessive inflammation together with improved vascular organization and potentially improved oxygen/nutrient support may better support the metabolic requirements of re-epithelialization and dermal reconstruction [44]. Histological examination also suggested improved dermal organization and a tendency toward appendage-like structure recovery in the CoPS-CPG-treated group, raising the possibility that CoPS-CPG may improve not only the rate but also the quality of repair [45,46]. Visible hair regrowth and the increased presence of follicular or adnexal-like structures in H&E sections were consistent with this interpretation. However, because this semi-quantitative analysis was based on H&E morphology and dedicated hair follicle lineage markers were not included, these findings should be considered suggestive rather than definitive evidence of hair follicle regeneration and warrant further validation in future studies. In this acute murine wound model, untreated control wounds would also be expected to progress toward spontaneous closure with sufficient time. Therefore, the present findings should be interpreted primarily as evidence that CoPS-CPG accelerated early-to-mid residual wound-area reduction and improved day-14 repair-associated tissue features, rather than as proof of superior final closure after all wounds have completely healed.

Several limitations should be acknowledged. First, the topical formulation was characterized by visual/spreadability observation, inversion testing, oscillatory rheological analysis, and 7-day pH/preliminary physical stability assessment. However, steady-shear viscosity, release kinetics, long-term storage stability, and batch-to-batch consistency remain to be further investigated. Second, the murine excisional wound model used here was not splinted; therefore, residual wound-area reduction may include contraction-mediated closure and should not be interpreted as direct evidence of re-epithelialization alone. Future studies using splinted wound models are needed to better distinguish re-epithelialization from contraction. Third, although CD206-, VEGF-, CD31-, α-SMA-, and HIF-1α-associated signals were altered after treatment, the present study remains correlative and does not identify a direct molecular receptor or signaling pathway through which CoPS acts. In addition, selective enzymatic hydrolysis was not performed; endo-levanase digestion followed by oligosaccharide profiling would provide complementary fragment-level evidence for the proposed levan-type architecture. Fourth, dose–response analysis, later post-wounding time points after complete closure, long-term scar-quality assessment, collagen-remodeling readouts, and direct perfusion measurements were not included. These issues should be addressed in future studies to define the therapeutic window and translational potential of CoPS more rigorously.

## 4. Materials and Methods

### 4.1. Plant Sources and Polysaccharide Preparation

**Scheme 1:**
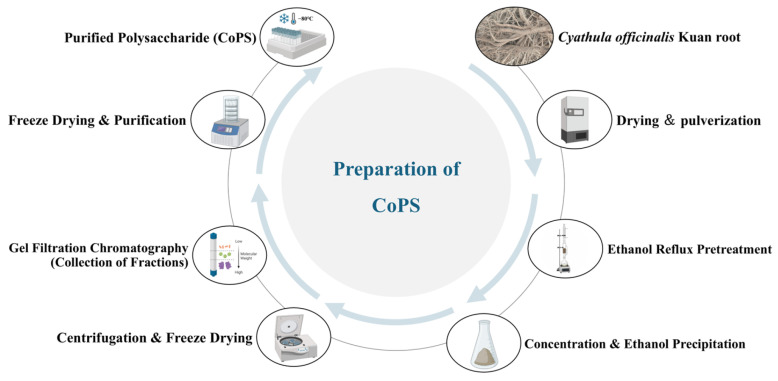
Workflow for extraction and purification of CoPS.

The roots of *C. officinalis* were collected from Ya’an, Sichuan Province, China. The extraction and purification flow chart of polysaccharides is shown in Scheme 1. After removal of surface impurities, the roots were dried at 105 °C for 30 min and then at 50 °C to constant weight, ground into powder, and stored in a dry environment until use. To remove low-molecular-weight lipophilic constituents and pigments, the powder (100 g) was refluxed with anhydrous ethanol at a solvent-to-material ratio of 20 mL g^−1^ for 1 h, and this procedure was repeated seven times. The residue was collected by filtration and dried to remove residual ethanol. The pretreated powder was then extracted with distilled water at a solid-to-liquid ratio of 1:20 (*w*/*v*) at 100 °C for 2 h, and extraction was repeated three times. The combined aqueous extracts were concentrated and precipitated with ethanol to a final concentration of 85% (*v*/*v*), followed by standing at 4 °C for 24 h. The precipitate was collected by centrifugation and freeze-dried to obtain the crude polysaccharides.

The crude polysaccharides were sequentially purified by DEAE-Sepharose Fast Flow column chromatography (4.6 cm × 60 cm) and Sepharose 6FF gel filtration chromatography (2.5 cm × 100 cm). The purified fraction was collected, freeze-dried, and used for subsequent structural characterization and bioassays. Total carbohydrate content was determined by the phenol–sulfuric acid method using glucose as the calibration standard. Protein content was determined by the Coomassie Brilliant Blue G-250 method using bovine serum albumin (BSA) as the standard. Total phenolic content was determined by the Folin–Ciocalteu method using gallic acid as the calibration standard. In all assays, absorbance was measured using a microplate reader, and the corresponding contents were calculated from the respective standard curves. All measurements were performed in triplicate.

### 4.2. Characterization of Polysaccharide Structure

#### 4.2.1. UV Spectral and X-Ray Diffraction Analysis

The UV-vis spectrum of the sample solution (5 mg/mL) was recorded using a multifunctional microplate reader (Multiskan GO, Thermo Fisher Scientific Oy, Vantaa, Finland) over a wavelength range of 200–1000 nm, with a scan interval of 1 nm. Pure water was used as the blank control, and measurements were conducted under identical conditions.

The crystallinity of the polysaccharide was assessed at room temperature using an X-ray diffractometer (XRD, Ultima IV, Rigaku Corporation, Tokyo, Japan), operated at 40 kV and 40 mA with Cu-Kα monochromatic radiation (wavelength: 0.15406 nm) in the 2θ range of 5–60°. The step size and scanning speed were set to 0.02° and 2°/min, respectively.

#### 4.2.2. Molecular Weight Determination

Polysaccharide fractions were dissolved in aqueous 0.1 M NaNO_3_ containing 0.02% (*w*/*v*) NaN_3_ to a final concentration of 1.0 mg mL^−1^, and the solutions were filtered through a 0.45 µm pore-size membrane before injection. For homogeneity and molecular-weight determination, size-exclusion chromatography coupled with multi-angle laser light scattering and refractive-index detection (SEC–MALLS–RI) was employed. The chromatographic system comprised two tandem columns (300 × 8 mm, Shodex OH-pak SB-805 and SB-803, Showa Denko K.K., Tokyo, Japan) maintained at 45 °C by a column heater (Sanshu Biotech Co., Ltd., Shanghai, China). The mobile phase was the same 0.1 M NaNO_3_/0.02% NaN_3_ aqueous solution, and the flow rate was set to 0.6 mL min^−1^. Eluate from the columns passed sequentially into a DAWN HELEOS-II laser photometer (Wyatt Technology, Santa Barbara, CA, USA) and an Optilab T-rEX refractive-index detector (Wyatt Technology Co., USA). The differential refractive index increment (dn/dc) of the polysaccharide fractions in this solvent system was determined as 0.141 mL g^−1^. Data acquisition and analysis were performed using ASTRA 6.1 (Wyatt Technology), and molecular-weight averages (*M*_w_ and *M*_n_) and dispersity (*M*_w_/*M*_n_) were exported to Excel for further processing.

#### 4.2.3. Monosaccharide Composition and FTIR Spectrum

Monosaccharide composition, including fructose, was determined by high-performance anion-exchange chromatography with pulsed amperometric detection (HPAEC–PAD). Trifluoroacetic acid (TFA) was obtained from ANPEL; sodium hydroxide, methanol, and sodium acetate trihydrate were obtained from Sigma-Aldrich (St. Louis, MO, USA). Ultrapure water was prepared in-house using a Milli-Q system (Millipore, Bedford, MA, USA). Approximately 5 mg of purified polysaccharide was hydrolyzed with 2 M TFA at 60 °C for 30 min in a sealed tube. The hydrolysate was dried under a gentle nitrogen stream, washed with methanol, and dried again; the methanol wash was repeated 2–3 times to remove residual acid. The residue was redissolved in deionized water and passed through a 0.22 μm microporous membrane before injection. Chromatographic analyses were conducted on a CarboPac PA-20 anion-exchange column (3 × 150 mm; Thermo Fisher Scientific, San Jose, CA, USA) coupled to a Dionex ICS 5000+ system equipped with PAD (Thermo Fisher Scientific, San Jose, CA, USA). The flow rate was 0.5 mL min^−1,^ and the injection volume was 5 μL. Eluents were as follows: A, water; B, 0.1 M NaOH; and C, 0.1 M NaOH with 0.2 M sodium acetate. The gradient (A:B:C, *v*/*v*/*v*) was programmed as follows: 95:5:0 at 0 min; 85:5:10 at 26 min; held at 85:5:10 to 42 min; 60:0:40 at 42.1 min; 60:40:0 at 52 min; returned to 95:5:0 at 52.1 min and held to 60 min. Data were acquired using the ICS 5000+ system and processed using Chromeleon 7.2 CDS (Thermo Scientific); quantitative results were exported in Excel format.

Fourier transform infrared (FTIR) spectroscopy was performed using a Nicolet iS10 FTIR spectrometer (Thermo Electron Scientific Instruments LLC, Madison, WI, USA). The instrument resolution was 4.00 cm^−1^, and the scanning range was 4000–400 cm^−1^. Data were collected with a 32-scan average, a sampling gain of 8.0, and a mirror velocity of 0.4747. Measurements were conducted with a DTGS KBr detector, a KBr beamsplitter, and an infrared light source. Polysaccharide samples were mixed with KBr powder and pressed into 1 mm thick pellets for analysis. The FTIR spectra were obtained in the range of 4000–400 cm^−1^ by Sanshu Biotech Co., Ltd. (Shanghai, China).

#### 4.2.4. Scanning Electron Microscopy (SEM) Analysis

Scanning electron microscopy (SEM; Merlin Compact, Carl Zeiss Microscopy GmbH, Jena, Germany) was used to examine the surface morphology of the polysaccharide sample. The samples were lyophilized before being coated with a thin gold layer and placed on a substrate for analysis. Imaging was performed under high vacuum at a voltage of 1.0 kV with magnifications of 1000× and 10,000×. The examination was carried out by Sanshu Biotech Co., Ltd. (Shanghai, China).

#### 4.2.5. Methylation Analysis

The polysaccharide sample was dissolved in DMSO, and methylation was carried out in a DMSO/NaOH medium with CH_3_I as the methylating agent. After completion of the methylation reaction, the permethylated products were hydrolyzed using 2 mol L^−1^ TFA at 60 °C for 1 h, followed by reduction with NaBD_4_ and subsequent acetylation with acetic anhydride at 100 °C for 2.5 h. The resulting acetylated derivatives were dissolved in chloroform and subjected to gas chromatography–mass spectrometry (GC–MS) analysis using an Agilent 6890A GC coupled to a 5977B MSD (Agilent Technologies, Santa Clara, CA, USA) equipped with a TG-200 capillary column (30 m × 0.25 mm × 0.25 µm; SGE Analytical Science Pty Ltd., Ringwood, VIC, Australia). High-purity helium served as the carrier gas at a split ratio of 10:1, and the injection volume was 1 µL. The GC oven was held at 150 °C for 2.0 min, ramped to 210 °C at 2 °C min^−1^ and held for 3 min, and then increased to 240 °C at 2 °C min^−1^ and held for 5 min. Mass spectrometric detection was performed in SCAN mode over an m/z range of 50–350.

#### 4.2.6. NMR Spectroscopy Analysis and Structural Characterization

For sample preparation, an appropriate amount of purified polysaccharide was dissolved in D_2_O to achieve a concentration of at least 40 mg/mL. The resulting solution (0.5 mL) was transferred into a 5 mm NMR tube for analysis. NMR spectra were recorded using a Bruker AVANCE NEO 500 MHz spectrometer (Bruker, Rheinstetten, Germany) at 25 °C. 1D spectra, including ^1^H NMR, ^13^C NMR, and DEPT-135, and 2D spectra, including COSY, TOCSY, HMBC, and HSQC, were obtained. Measurements were performed using a liquid probe (QXI ^1^H/^31^P/^13^C/^15^N 5 mm) with a Z-gradient (ATM Acc). The technical parameters were as follows: the signal-to-noise ratio (SNR) for ^1^H was 888, the resolution for ^1^H was 0.32 Hz (rotating), and the SNR and resolution for ^13^C were 328 and 0.1 Hz, respectively. Data acquisition and processing were performed by Sanshu Biotech Co., Ltd. (Shanghai, China).

### 4.3. Preparation of the CoPS-Containing Topical Formulation

A Carbomer/alginate-based semisolid formulation was prepared as the topical delivery vehicle for CoPS. Briefly, Carbomer powder (2 g) was gradually dispersed in 50 mL of ultrapure water under vigorous stirring until fully hydrated. The dispersion was allowed to swell in a 40–60 °C water bath for 10–30 min, followed by pH adjustment to 6.0–7.0 using NaOH to obtain a transparent and viscous Carbomer gel. CoPS (0.5 g) was pre-dispersed in a small volume of ultrapure water and added to the Carbomer gel to obtain a CoPS concentration of 1% (*w*/*v*) in the precursor mixture, with continuous stirring to ensure homogeneous distribution. Glycerol (5 g) and sodium alginate (1.0 g) were then incorporated, and the mixture was stirred thoroughly until a homogeneous semisolid formulation was obtained. The CoPS-containing formulation was designated CoPS-CPG. The corresponding vehicle formulation, prepared in the same manner but without CoPS, was designated Blank-CPG. Before subsequent experiments, the formulations were allowed to stand overnight at room temperature, and entrapped air bubbles were removed by vacuum degassing or standing.

### 4.4. Rheological Characterization and Short-Term pH/Physical Stability Assessment

The rheological behavior of CoPS-CPG was evaluated using a Thermo Scientific HAAKE MARS 40 rotational rheometer (Thermo Fisher Scientific, USA). Oscillatory frequency sweep measurements were performed at room temperature (approximately 25 °C) over a frequency range of 0.1–10 Hz with a fixed strain of 1%. The storage modulus (G′), loss modulus (G″), and complex viscosity |η*| were recorded.

For short-term pH and preliminary physical stability assessment, freshly prepared Blank-CPG and CoPS-CPG formulations were stored at room temperature (approximately 25 °C). pH values were measured on days 0, 1, 3, 5, and 7 using a calibrated pH meter. Appearance, visible instability, and semisolid consistency were recorded at each time point. Each pH measurement was performed in triplicate, and data are presented as mean ± SD.

### 4.5. Cytocompatibility Assessment

RAW264.7 murine macrophages (TCM13) and HaCaT human keratinocytes (GNHu64) were obtained from the Cell Bank of the Chinese Academy of Sciences, Shanghai, China. Both cell lines were cultured in DMEM supplemented with 10% fetal bovine serum at 37 °C in a humidified atmosphere containing 5% CO_2_. Cells in the logarithmic growth phase were used for all in vitro experiments.

The cytocompatibility of Blank-CPG and CoPS-CPG was evaluated by a live/dead viability assay using RAW264.7 macrophages and HaCaT keratinocytes. Formulation-coated 24-well plates were prepared in advance and sterilized by ultraviolet irradiation before cell seeding. RAW264.7 and HaCaT cells were seeded at densities of 1.0 × 10^5^ and 8.0 × 10^4^ cells per well, respectively. Cells cultured on tissue culture polystyrene without formulation served as the control. At the indicated time points, cells were washed with PBS and incubated with calcein-AM (2 μM) and propidium iodide (PI, 4 μM) for 15 min in the dark. Fluorescence images were acquired using a fluorescence microscope, and cell viability was quantified from at least five randomly selected fields using ImageJ software (version 1.54g; National Institutes of Health, Bethesda, MD, USA). Live cells were identified by green fluorescence and dead cells by red fluorescence.

### 4.6. Cell Migration Assay

The effect of the CoPS-containing formulation on keratinocyte migration was evaluated using an in vitro scratch assay. HaCaT cells were seeded in 6-well plates at a density of 3.0 × 10^5^ cells per well and cultured in complete medium under standard conditions (37 °C, 5% CO_2_) until 90–100% confluence was reached. A uniform linear scratch was generated across the cell monolayer using a sterile 200 μL pipette tip held perpendicular to the plate surface. Detached cells and debris were removed by washing twice with phosphate-buffered saline (PBS). Cells were then incubated in serum-reduced medium (1% fetal bovine serum) containing extracts prepared from Blank-CPG or CoPS-CPG, or in vehicle medium alone (control), to minimize the contribution of cell proliferation to scratch closure. Images were acquired immediately after scratching (0 h) and again at 12 and 24 h using an inverted microscope at identical positions in each well. For quantitative analysis, the residual scratch area was measured using ImageJ and normalized to the initial scratch area at 0 h for each group.

### 4.7. Animal Model Establishment of Skin Injury

All animal procedures were approved by the Sichuan Agricultural University Institutional Animal Care and Use Committee (approval no. 20250467, approved on 15 July 2025) and were conducted in accordance with institutional guidelines, the ARRIVE recommendations, and the Guide for the Care and Use of Laboratory Animals. Healthy adult male C57BL/6 mice (6–8 weeks old, 20–25 g) were obtained from Beijing Huafukang Biotechnology Co., Ltd. (Beijing, China) and housed under specific pathogen-free (SPF) conditions at a controlled ambient temperature of 22 ± 2 °C, relative humidity of 50 ± 10%, and a 12 h light/12 h dark cycle. Animals had ad libitum access to standard chow and water, with daily environmental condition monitoring. Mice were acclimated to the facility for at least 7 days before the experiment.

For surgery, anesthesia was induced with ketamine (100 mg/kg) and xylazine (10 mg/kg) administered intraperitoneally, and buprenorphine (0.05–0.1 mg/kg, subcutaneously) was used for preoperative analgesia. A 6 mm full-thickness excisional wound was created on the dorsum, and the surgical procedure was performed aseptically. Animals were randomly assigned to groups using a computer-generated randomization schedule. Wound-area quantification and histological/immunostaining analyses were performed by investigators blinded to treatment allocation. For topical treatment, 50 μL of formulation was applied to each wound immediately after injury and once daily thereafter. Wound areas were documented at predefined time points (days 0, 3, 5, 7, 10, and 14). Wound area was quantified from digital images using ImageJ, and residual wound area was calculated as At/A0 × 100%, where A0 is the wound area on day 0 and At is the wound area at the indicated time point.

For longitudinal residual wound-area analysis, *n* = 6 animals per group were followed over time. For day-4 and day-14 tissue analyses, *n* = 6 animals per group per time point were collected from independent animals. Exclusion criteria included anesthesia or modeling failure, wound infection, severe scratching or biting of the wound, loss of treatment, or reaching humane endpoints (e.g., >20% body weight loss or signs of distress). All efforts were made to minimize animal suffering and reduce the number of animals used in accordance with the 3Rs principles.

#### 4.7.1. Hematoxylin and Eosin (H&E) Staining

Wound tissue samples collected at the indicated time points were fixed in 4% paraformaldehyde at room temperature for 24 h, dehydrated through a graded ethanol series, cleared in xylene, and embedded in paraffin. Paraffin-embedded tissues were sectioned at 4 μm thickness using a rotary microtome. The sections were deparaffinized in xylene, rehydrated through graded ethanol to distilled water, and stained with hematoxylin and eosin according to standard procedures. After staining, the sections were dehydrated, cleared, and mounted with neutral resin for histological evaluation. For semi-quantitative analysis, hair follicle-like/adnexal-like structures were counted in day-14 H&E-stained sections within the regenerated wound area. Normal skin outside the wound bed and obvious tissue artifacts were excluded. For each animal, three to five non-overlapping fields of view were analyzed at the same magnification, and the average number of follicle/adnexal-like structures per field was calculated. Quantification was performed in a blinded manner, and data are presented as mean ± SD.

#### 4.7.2. Preparation of Wound Tissue Homogenates and ELISA

On days 4 and 14 post-wounding, mice were euthanized, and wound tissues were harvested. A standardized full-thickness biopsy, including the wound bed and a 1–2 mm margin of surrounding skin, was excised, briefly rinsed with ice-cold PBS to remove residual blood, and weighed. Tissues were homogenized in ice-cold PBS supplemented with protease inhibitor cocktail at a ratio of 1:10 (*w*/*v*). The homogenates were centrifuged at 10,000× *g* for 10 min at 4 °C, and the supernatants were collected for cytokine analysis. Total protein concentration was determined using a BCA protein assay. The levels of IL-6, IL-1β, TNF-α, and IL-10 were measured using commercial ELISA kits (Wuhan Servicebio Technology Co., Ltd., Wuhan, China) according to the manufacturer’s instructions. Cytokine levels were normalized to total protein content and expressed as pg/mg total protein.

#### 4.7.3. Immunohistochemistry (IHC)

Paraffin-embedded tissue sections (4 μm) were deparaffinized and rehydrated as described above. Antigen retrieval was performed in citrate buffer (pH 6.0; Servicebio antigen retrieval solution), followed by cooling to room temperature. Endogenous peroxidase activity was blocked with 3% H_2_O_2_ for 10 min at room temperature. After washing, sections were blocked with 10% normal goat serum for 30 min at room temperature and then incubated overnight at 4 °C with primary antibodies against TNF-α (Servicebio, GB115702, 1:400) or VEGF (Servicebio, GB15165, 1:200). After PBS washes, sections were incubated with an HRP-conjugated goat anti-rabbit secondary antibody (Servicebio) and visualized using a DAB substrate kit (Servicebio, G1212). Nuclei were counterstained with hematoxylin, and the sections were dehydrated, cleared, and mounted. Bright-field images were captured using an optical microscope (Olympus IX73, Olympus, Japan), and the positive staining area and/or staining intensity were quantified using ImageJ.

#### 4.7.4. Immunofluorescence (IF) Staining

Paraffin-embedded tissue sections were deparaffinized, rehydrated, and subjected to heat-mediated antigen retrieval in citrate buffer (pH 6.0). After blocking with 10% normal goat serum (Servicebio) for 30 min at room temperature, sections were incubated overnight at 4 °C with the indicated primary antibodies. Following PBS washes, sections were incubated with species-matched fluorophore-conjugated secondary antibodies (Servicebio) for 1 h at room temperature in the dark. Nuclei were counterstained with DAPI (Servicebio, G1012), and slides were mounted with antifade mounting medium (Servicebio). Fluorescence images were acquired using a confocal microscope (ZEISS LSM 980, Carl Zeiss, Germany).

For double immunofluorescence staining, CD31 (Servicebio, GB120005, 1:200) and α-SMA (Servicebio, GB111364, 1:400) were used to evaluate vascular structures and perivascular coverage, with CD31 visualized in the red channel and α-SMA in the green channel. CD206 (Servicebio, GB113497) and CD68 (Thermo Fisher/eBioscience, clone FA-11, Cat. 14-0681-82) were used to assess macrophage-associated signals, with CD206 visualized in the red channel and CD68 in the green channel. For single immunofluorescence staining, Ki67 (Servicebio, GB111499) was detected in the red channel. HIF-1α was detected using a FITC-conjugated antibody (Thermo Fisher, MA5-45251, clone ESEE122) and imaged in the green channel, with DAPI used as the nuclear counterstain.

#### 4.7.5. Statistical Analysis

Data are presented as mean ± SD. Statistical analyses were performed using GraphPad Prism 10. Normality was assessed using the Shapiro–Wilk test. Longitudinal residual wound-area data obtained from the same animals over time were analyzed using two-way repeated-measures ANOVA followed by Tukey’s multiple-comparison test; when missing values occurred, a mixed-effects model was used. Comparisons among three groups at a single time point were analyzed using one-way ANOVA followed by Tukey’s post hoc test. For comparisons between two groups, an unpaired two-tailed Student’s t-test was used when normality assumptions were met. A value of *p* < 0.05 was considered statistically significant. Statistical annotations are denoted as ns, not significant; * *p* < 0.05, ** *p* < 0.01, and *** *p* < 0.001. Exact *n* values and statistical tests are indicated in the corresponding figure legends.

## 5. Conclusions

In this study, a homogeneous water-soluble polysaccharide fraction (CoPS) was isolated from the roots of *C. officinalis* and structurally identified as a branched levan-type fructan with a β-(2→6)-dominated fructofuranosyl backbone and β-(2→1)-linked branching motifs. Biological evaluation using a topical Carbomer/alginate formulation showed that CoPS-CPG had good cytocompatibility and promoted wound-healing-related responses. In a murine full-thickness cutaneous wound model, topical administration of the CoPS-containing formulation accelerated residual wound-area reduction and improved histological tissue reconstruction during the observation period. These beneficial effects were accompanied by a reduced inflammatory burden, enrichment of CD206-associated macrophage features, increased CD31-associated vascular structures, improved α-SMA-associated perivascular coverage, and lower late-stage HIF-1α signal. Together, these findings suggest that this structurally defined fructan contributes to a repair-supportive immune microenvironment and improved vascular organization-associated tissue repair during cutaneous healing. Overall, this study provides structural insight into a plant-derived levan-type fructan from *C. officinalis* and supports its potential as a bioactive polysaccharide for wound-repair applications.

## Figures and Tables

**Table 1:** Linkage analysis of CoPS by methylation followed by GC–MS.

Derivative Name	RT	Mw	Molar Ratio (%)	Glycosidic Linkage
2,5-di-O-acetyl-(2-deuterio)-1,3,4,6-tetra-O-methyl hexitols (mannitol,glucitol)	11.430	323	17.00	t-Fruf
2,5-di-O-acetyl-(2-deuterio)-1,3,4,6-tetra-O-methyl hexitols (mannitol,glucitol)	11.588	323	8.99	t-Fruf
Subtotal			25.99	t-Fruf
1,5-di-O-acetyl-2,3,4,6-tetra-O-methyl glucitol	13.593	323	10.46	t-Glcp
Subtotal			10.46	t-Glcp
2,5,6-tri-O-acetyl-(2-deuterio)-1,3,4-tri-O-methyl hexitols (mannitol,glucitol)	18.246	351	25.56	6-Fruf
2,5,6-tri-O-acetyl-(2-deuterio)-1,3,4-tri-O-methyl hexitols (mannitol,glucitol)	18.574	351	18.67	6-Fruf
Subtotal			44.23	6-Fruf
1,2,5-tri-O-acetyl-(2-deuterio)-3,4,6-tri-O-methyl hexitols (mannitol,glucitol)	18.308	351	7.67	1-Fruf
Subtotal			7.67	1-Fruf
1,2,5,6-tetra-O-acetyl-(2-deuterio)-3,4-di-O-methyl hexitols (mannitol,glucitol)	26.081	379	7.20	1,6-Fruf
1,2,5,6-tetra-O-acetyl-(2-deuterio)-3,4-di-O-methyl hexitols (mannitol,glucitol)	26.287	379	4.44	1,6-Fruf
Subtotal			11.64	1,6-Fruf
Total (Fru-derived)			89.53	
Total (Glc-derived)			10.46	
Grand total			99.99	

Note: Fructose (a ketose) can be reduced to mannitol- and glucitol-type derivatives during the PMAA workflow, and therefore, a given fructose linkage class may appear as paired GC peaks; linkage-type proportions are interpreted by summing peak areas of the same assigned linkage.

**Table 2:** ^1^H and ^13^C NMR chemical-shift assignments of the glycosyl residues in CoPS.

Code	Glycosyl Residues	Chemical Shifts (ppm)					
		H1/C1	H2/C2	H3/C3	H4/C4	H5/C5	H6/C6
A	→6)-β-D-Fruf-(2→	3.68, 3.61	/	4.12	4.04	3.88	3.82, 3.50
		59.91	104.17	76.45	74.56	80.23	63.23
B	β-D-Fruf-(2→	3.76, 3.69	/	4.17	4.06	3.80	3.77, 3.58
		60.33	103.65	76.65	75.14	81.06	62.44
C	→1,6)-β-D-Fruf-(2→	3.77, 3.67	/	4.10	4.03	3.87	3.87, 3.68
		60.25	103.77	76.26	74.35	80.18	63.15
D	α-D-Glcp-(1→	5.33	3.47	3.68	3.39	3.76	3.80
		92.47	71.18	72.65	69.37	72.34	60.06
E	→1)-β-D-Fruf-(2→	3.81, 3.62	/	4.16	4.05	3.79	3.74, 3.58
		60.09	103.17	76.96	75.31	80.98	62.20

Note: “/” indicates unmeasured data.

## Data Availability

The original contributions presented in this study are included in the article. Further inquiries can be directed to the corresponding authors.

## Supplementary Materials

The following supporting information can be downloaded at https://www.mdpi.com/article/10.3390/molecules31111981/s1: Figure S1: GC–MS total ion chromatogram (TIC) of partially methylated alditol acetate derivatives obtained from methylated CoPS; Figure S2: COSY spectrum of CoPS showing through-bond proton–proton correlations used for residue-level assignment; Figure S3: TOCSY spectrum of CoPS showing extended through-residue proton correlations in the overlapped proton region.; Table S1: Preliminary short-term pH and physical stability during storage; Table S2: Semi-quantitative count of hair follicle-like/adnexal-like structures in day-14 H&E-stained wound sections.

## Abbreviations

The following abbreviations are used in this manuscript:

ANOVA	Analysis of Variance
BSA	Bovine Serum Albumin
CD68	Cluster of Differentiation 68
CD206	Cluster of Differentiation 206
COSY	Correlation Spectroscopy
DEPT-135	Distortionless Enhancement by Polarization Transfer (135°)
DMEM	Dulbecco’s Modified Eagle Medium
DMSO	Dimethyl Sulfoxide
ELISA	Enzyme-Linked Immunosorbent Assay
FTIR	Fourier Transform Infrared Spectroscopy
GC–MS	Gas Chromatography–Mass Spectrometry
H&E	Hematoxylin and Eosin
HIF-1α	Hypoxia-Inducible Factor 1 Alpha
HPAEC–PAD	High-Performance Anion-Exchange Chromatography with Pulsed Amperometric Detection
IF	Immunofluorescence
IL-10	Interleukin 10
IL-6	Interleukin 6
IR	Infrared
NMR	Nuclear Magnetic Resonance
PBS	Phosphate-Buffered Saline
SEM	Scanning Electron Microscopy
SEC–MALLS–RI	Size-Exclusion Chromatography–Multi-Angle Laser Light Scattering–Refractive Index
SPF	Specific Pathogen-Free
TNF-α	Tumor Necrosis Factor Alpha
VEGF	Vascular Endothelial Growth Factor
XRD	X-Ray Diffraction
