# Supplementary material for: Structure and Wound-Healing Activity of a Branched Levan-Type Fructan from Cyathula officinalis Roots
Source: Molecules. 2026 Jun 5;31(11):1981. doi: 10.3390/molecules31111981 (PMC13258527; doi:10.3390/molecules31111981)
Supplement: Supplementary file 1 [file molecules-31-01981-s001.zip › molecules-4301352-supplementary.pdf]

Table S1. Preliminary short-term pH and physical stability during storage.

**Formulation**	Time	pH	Appearance	Visible instability	Consistency
Blank-CPG	0 d	6.51 ± 0.04	Homogeneous	None	Semisolid
Blank-CPG	1 d	6.50 ± 0.02	Homogeneous	None	Semisolid
Blank-CPG	3 d	6.48 ± 0.03	Homogeneous	None	Semisolid
Blank-CPG	5 d	6.49 ± 0.05	Homogeneous	None	Semisolid
Blank-CPG	7 d	6.48 ± 0.03	Homogeneous	None	Semisolid
CoPS-CPG	0 d	6.50 ± 0.04	Homogeneous	None	Semisolid
CoPS-CPG	1 d	6.51 ± 0.02	Homogeneous	None	Semisolid
CoPS-CPG	3 d	6.48 ± 0.03	Homogeneous	None	Semisolid
CoPS-CPG	5 d	6.49 ± 0.04	Homogeneous	None	Semisolid
CoPS-CPG	7 d	6.47 ± 0.03	Homogeneous	None	Semisolid

Data are presented as mean ± SD, n = 3. Visible instability refers to phase separation, precipitation, discoloration, or obvious loss of semisolid consistency.

Table S2. Semi-quantitative count of hair follicle-like/adnexal-like structures in day-14 H&E-stained wound sections

**Group**	Hair follicle-like/adnexal-like structures per field
Control	0.40 ± 0.55
Blank-CPG	0.60 ± 0.55
CoPS-CPG	2.80 ± 0.84

Data are presented as mean ± SD. Counts were performed in day-14 H&E-stained sections within the regenerated wound area. Normal skin outside the wound bed and obvious tissue artifacts were excluded.

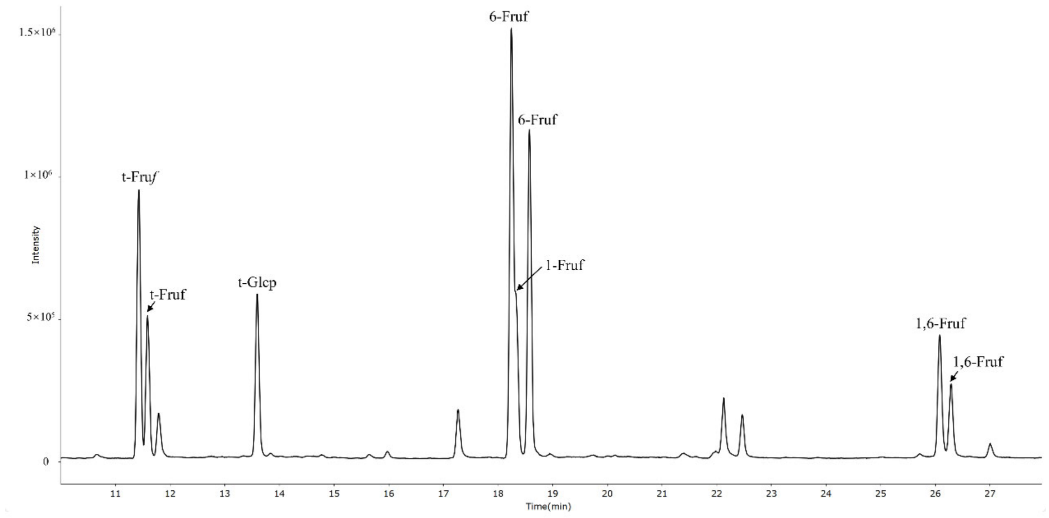

Figure S1. GC–MS total ion chromatogram (TIC) of partially methylated alditol acetate derivatives obtained from methylated CoPS.

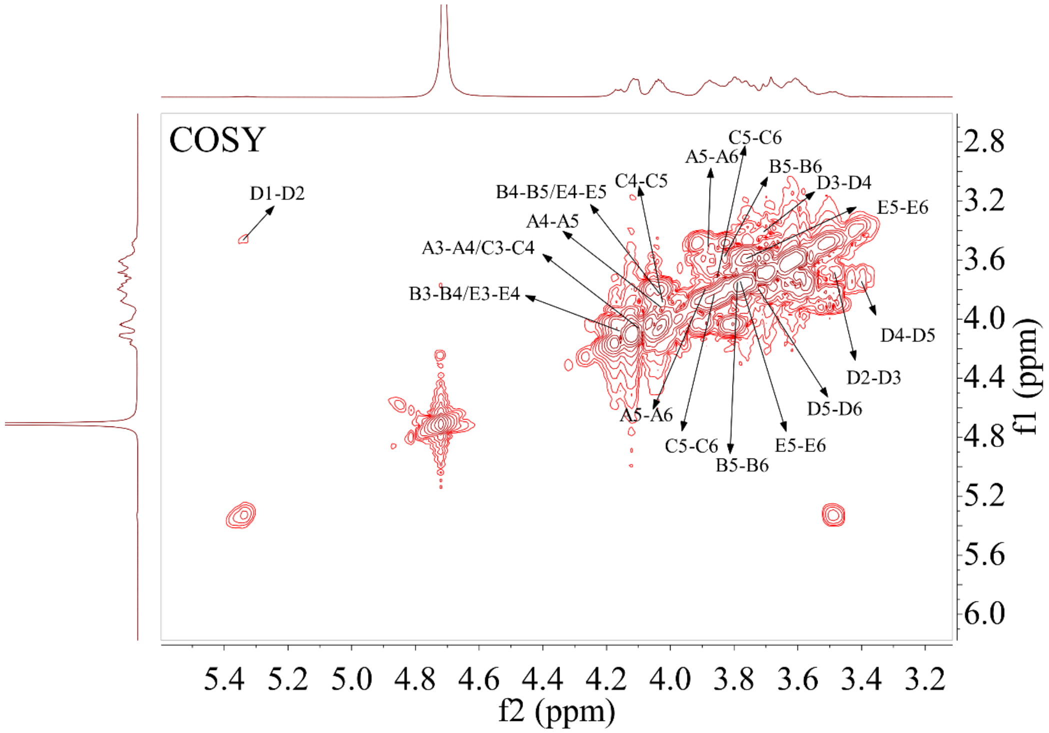

Figure S2. COSY spectrum of CoPS showing through-bond proton–proton correlations used for residue-level assignment.

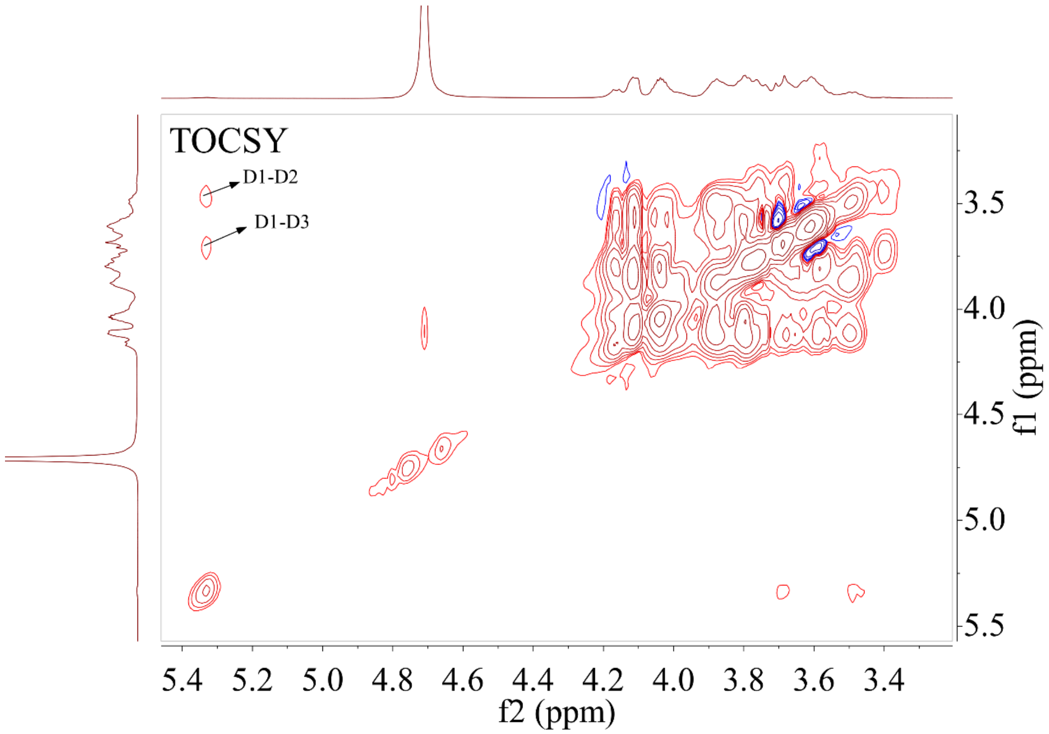

Figure S3. TOCSY spectrum of CoPS showing extended through-residue proton correlations in the overlapped proton region.
